# Outer Retinal Layers' Thickness Changes in relation to Age and Choroidal Thickness in Normal Eyes

**DOI:** 10.1155/2019/1698967

**Published:** 2019-07-31

**Authors:** Mona Kamal Abdellatif, Yasser Abdelmaguid Mohamed Elzankalony, Ahmed Abdelmonsef Abdelhamid Ebeid, Weam Mohamed Ebeid

**Affiliations:** Department of Ophthalmology, Faculty of Medicine, Ain Shams University, Cairo, Egypt

## Abstract

**Purpose:**

To identify and correlate age-related changes in outer retinal layers' thickness and choroidal thickness (CT) in the normal eyes using spectral-domain optical coherence tomography (SD-OCT) and to investigate factors affecting these changes.

**Study Design:**

Observational cross-sectional study.

**Subjects and Methods:**

We studied 125 healthy Egyptians between 20 and 79 years old. Patients were divided into 3 groups: group 1 (20–40 years), group 2 (40–60 years), and group 3 (>60 years). All patients had full ophthalmic examination. SD-OCT was done to measure the 9 ETDRS macular grid sectors of retinal pigment epithelium and photoreceptor outer segment (RPE-OS), outer nuclear layer and photoreceptor inner segment (ONL-IS), and choroidal thickness (CT) (by enhanced depth imaging).

**Results:**

RPE-OS was significantly thinner in group 3 than in the other 2 groups (central: *P* < 0.001). Moreover, the 3 groups were significantly different from each other regarding the CT (central: *P* < 0.001); significant thinning was noticed in the choroid with age. The 3 groups did not show significant difference concerning the ONL-IS thickness. RPE-OS and CT showed statistically significant negative correlation with age (central RPE-OS: *r* = −0 C.345, *P* < 0.001, and central CT: *r* = −0.725, *P* < 0.001) while ONL-IS showed statistically nonsignificant correlation with age (central ONL-IS: *r* = −0.08, *P*=0.376). Multiple regression analysis revealed that the most important determinant of central 1 mm RPE-OS thickness in this study was age (*β* = −0.087, *P*=0.010) rather than choroidal thinning (*β* = 0.001, *P*=0.879).

**Conclusion:**

RPE-OS layer thickness shows significant thinning with increasing age, and with decrease in CT, however, age is the most determinant factor of this thinning.

## 1. Introduction

The new optical coherence tomography (OCT) devices provide high-resolution images of the retinal layers [1, 2]. This allows precise segmentation of individual retinal layers and provides thickness maps that could help in studying diseases affecting specific retinal layers [3].

Recently, the introduction of the enhanced depth imaging (EDI) technique to spectral-domain OCT by Spaide has added the advantage of detailed choroidal imaging [4].

Previous reports have indicated that total retinal thickness shows variations with age, but it is not well understood whether the differences in total retinal thickness may be explained by variations in thickness of the inner or outer retinal layers [5].

Earlier histological studies have suggested that there is a decrease in cone pigment with age [6], which indicates a loss and displacement of photoreceptors with age [7, 8].

Moreover, many studies have proved significant choroidal thinning with age [5, 9, 10], which could be of great importance since the outer retinal layers do not have a vascular network and rely on choriocapillaris, which exhibits a slow blood velocity, in supplying the retinal pigment epithelium (RPE) and outer retina with oxygen and nutrients, thus maintaining normal retinal function [11, 12].

Accordingly, variation in outer retinal layers' thickness with age could be either due to vascular insufficiency as a result of choroidal thickness change or direct effect due to the aging process itself [6].

Selective outer retinal degeneration (photoreceptor cells) occurs in various retinal degenerative processes such as age-related macular degeneration (AMD) and hereditary retinal dystrophies, and different therapeutic strategies have been proposed to slow the progression of photoreceptor cell loss in these cases [2]. Hence, studying the different factors that may affect photoreceptor thickness in normal eyes (including age) may help to understand the pathology and treatment response of these conditions.

The aim of our study was to identify and correlate age-related changes in outer retinal layers' thickness and choroidal thickness in normal eyes using spectral-domain optical coherence tomography (SD-OCT) and to investigate factors affecting these changes.

## 2. Patients and Methods

This cross-sectional observational clinical study was conducted between July 2018 and February 2019 in the Ophthalmology Department, Ain Shams University. It included 125 eyes of 125 healthy Egyptian individuals, and their ages ranged from 20 to 79 years.

The study was approved by the Research Ethical Committee at the Faculty of Medicine-Ain Shams University, with informed consent obtained from every patient.

All patients had full ophthalmic examination including best-corrected visual acuity testing with Snellen's charts, refraction, slit lamp biomicroscopy, dilated fundus ophthalmoscopy, axial length measurement using an A-scan device (Echo Scan US-800, Nidek, Aichi, Japan), and intraocular pressure measurement by using a Goldmann applanation tonometer at the outpatient clinic of the Ophthalmology Department of Ain Shams University. Only the right eye of every patient was examined.

Exclusion criteria included patients with systemic diseases that would affect the retina or choroid as diabetes, hypertension, renal diseases, infections, autoimmune diseases, and degenerative neuro-ophthalmological diseases. Patients with any known ocular pathology and history of previous ocular surgery, patients with glaucoma or intraocular pressure >18 mmHg, and patients with refractive error >+4 or <−4 were all excluded from the study.

## 3. Spectral-Domain Optical Coherence Tomography (SD-OCT)

**Figure 1:**
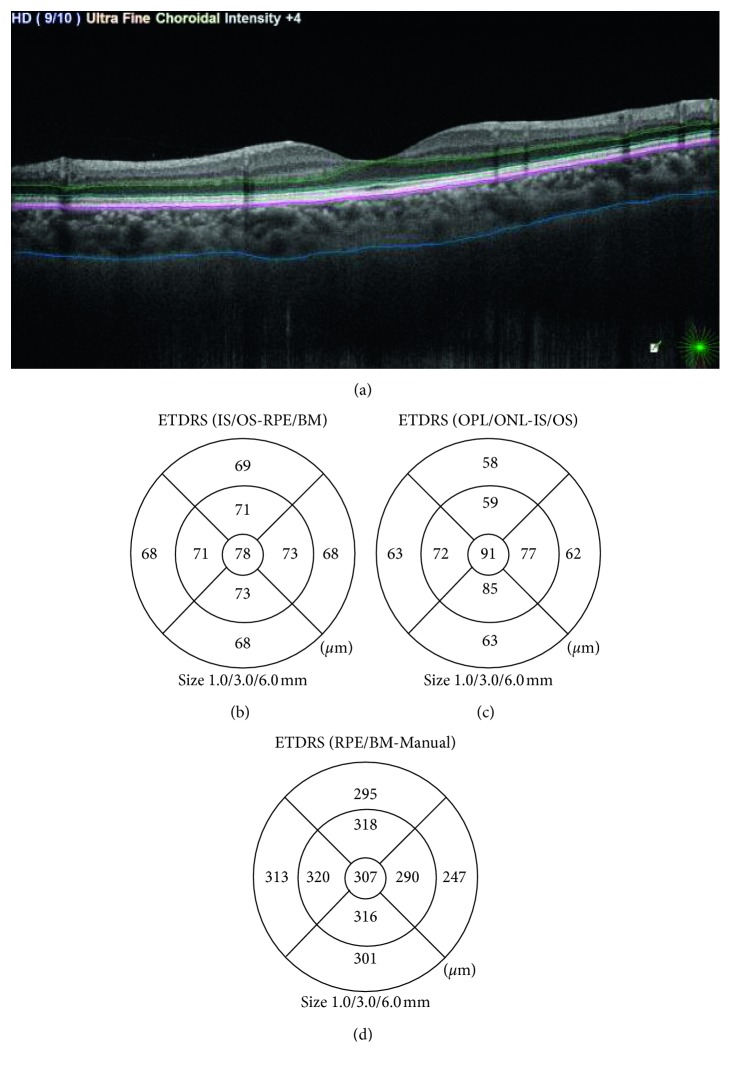
(a) OCT radial scan. ETDRS map: (b) retinal pigment epithelium and photoreceptor outer segment (RPE-OS) layer thickness (extending from the inner segment/outer segment (IS/OS) layer to the outer surface of RPE); (c) the outer nuclear layer and photoreceptor inner segment (ONL-IS) thickness (measured from the interface between the outer plexiform layer (OPL) and the outer nuclear layer (ONL) extending to the IS/OS layer); (d) choroidal thickness (CT) measured from the outer surface of RPE to the manually drawn sclerochoroidal interface.

Optical coherence tomography was performed by using the Nidek RS-3,000 Advance SD-OCT (Retinascan RS-3000 Advance, Nidek Co. Ltd., Gamagori, Japan), with scan speed 53,000 A-scan/s. All patients were examined between 10 am and 12 pm after mydriasis using Mydriacyl 0.5% eye drops. Radial lines scan was used to measure the retinal pigment epithelium and photoreceptor outer segment (RPE-OS) layer thickness (extending from the inner segment/outer segment (IS/OS) layer to the outer surface of RPE) and the outer nuclear layer and photoreceptor inner segment (ONL-IS) thickness (measured from the interface between the outer plexiform layer (OPL) and the outer nuclear layer (ONL) extending to the IS/OS layer). Macular thickness measurements were derived from the software (NAVIS-EX Image Filing software, RS-3,000 OCT, NIDEK, Gamagori, Japan) provided by the manufacturer; no manual manipulation of OCT data was performed in the calculation. ETDRS chart was obtained, and the 9 ETDRS subfields were measured. The ETDRS map standard retinal subﬁelds are central, inner (superior, inferior, nasal, and temporal), and outer (superior, inferior, nasal, and temporal). The central subﬁeld is bounded by the innermost 1 mm diameter circle. The inner and outer subﬁelds are bounded by the 3 mm and 6 mm diameter circles, respectively (Figure 1).

**Figure 2:**
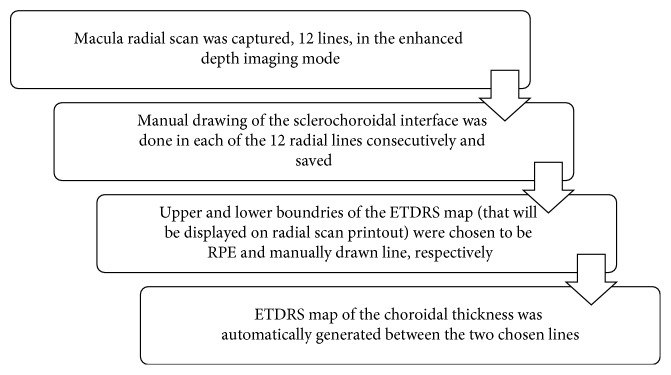
Schematic diagram showing the different steps performed to obtain the choroidal thickness map.

The choroid was imaged using the enhanced depth imaging technique (EDI) by positioning the SD-OCT closer to the eye to create an inverted image bringing the choroid to the zero delay line, which is the point of maximum sensitivity on SD-OCT. The image colors were made white on black to make the interface between the choroid and the sclera more distinct. The sclerochoroidal interface was drawn in the twelve radial lines manually. Choroidal thickness (CT) was measured from the outer surface of RPE to the manually drawn line. A CT map was automatically obtained with an ETDRS chart including the central CT in the innermost 1 mm circle and other ETDRS subfields (Figure 2). Drawing sclerochoroidal interface was performed by two independent expert investigators, and the average was taken. Only OCT scans with good signal quality were included (signal-to-noise ratio ≥7).

## 4. Statistical Analysis

All data analyses were performed using the Statistical Package for Social Sciences version 16.0 (SPSS© v. 16.0, SPSS Inc., Chicago, IL, USA). Quantitative data were presented as mean ± standard deviation (SD). Multiple group means were compared using the ANOVA test. Tukey's honestly significant difference (HSD) post hoc test was performed if an overall significance was found. Linear correlation coefficient was used for detection of correlation between two quantitative variables in one group. Regression analyses were done to assess the different factors that can affect ONL-IS and RPE-OS thickness in patients. The reliability and reproducibility of manual choroidal thickness measurements between the two investigators were measured using the interclass correlation coefficient (ICC). *P* values were considered statistically significant if <0.05.

## 5. Results

This study included 125 participants, with age ranged between 20 and 79 years old, with mean 47.32 ± 15.68 years old. Subjects were divided into 3 age groups: group 1 (45 participants, 20–40 years old), group 2 (43 participants, 40–60 years old), and group 3 (37 participants, age >60 years). There were 58 males (46.4%) and 67 females (53.6%) (Table 1).

Central 1 mm thickness as well as other 8 ETDRS subfields of the RPE-OS, the ONL-IS, and the choroid was measured in the three groups as shown in Table 2.

Interclass correlation coefficients (ICC) for CT measurements between the two investigators in the central 1 mm and the 8 ETDRS subfields (upper, lower, nasal and temporal quadrants in the inner and outer rings) were 0.994, 0.985, 0.964, 0.984, 0.988, 0.979, 0.982, 0.987, and 0.986 consecutively, denoting excellent reliability of manual CT measurements.

On comparing the 3 groups, central RPE-OS as well as other 8 ETDRS RPE-OS subfields was significantly thinner in group 3 than in the other 2 groups (*P* < 0.001). Moreover, a progressive significant choroidal thinning was evident in the 9 ETDRS subfields among the 3 groups along with aging (*P* < 0.001). The 3 groups did not show significant difference concerning the ONL-IS thickness (Table 2).

The nine ETDRS subfields of RPE-OS and CT showed statistically significant negative correlation with age (central RPE-OS: *r* = −0 C.345, *P* < 0.001, and central T: *r* = −0.725, *P* < 0.001), while ETDRS subfields of ONL-IS did not show any significant correlation with age except the inner temporal subfield (central ONL-IS: *P*=0.376) (Table 3).

RPE-OS showed positive statistically significant correlation with CT in all sectors of the ETDRS (central subfield: *r* = 0.273, *P*=0.002); however, ONL-IS showed insignificant correlation with CT (central subfield: *P*=0.409). No statistically significant correlation was found either with refraction or with axial length or gender in the three layers (Table 3).

Multiple regression analysis was carried out to detect factors more likely associated with thinning of RPE-OS layer. It revealed that the most important determinant of central 1 mm RPE-OS thickness in this study was age (*β* = −0.087, *P*=0.010) rather than choroidal thinning (*β* = 0.001, *P*=0.879) (Table 4).

Regarding other RPE-OS layer ETDRS sectors, regression analysis revealed that upper and lower zones thickness showed significant regression with age rather than CT (Table 4).

## 6. Discussion

New OCT technology has provided valuable quantitative information that could greatly enhance our understanding of outer retinal layers' diseases and allow clinicians to confirm their improvement or progression [13, 14]. Moreover, OCT may aid in evaluating the efficacy of new therapies by quantifying the photoreceptor cell layer in a reliable and reproducible way [2, 15]. For instance, previous studies using ultrahigh resolution OCT showed that the severity of photoreceptor loss is associated with visual loss in retinitis pigmentosa, [13] while other studies using SD-OCT showed that by measuring the thickness of the outer nuclear layer in the fovea, we can predict visual prognosis in retinal diseases such as central serous chorioretinopathy [16], polypoidal choroidal vasculopathy [17], and epiretinal membrane [15].

Earlier studies using SD-OCT [18–21] in healthy eyes reported changes in the macular profile in relation to factors such as age, sex, and axial length [15]. Thickness of different retinal layers has shown variation according to age in variable studies as well [8, 14, 21, 22]. Studying this expected variation in individual retinal layers using the new OCT technology would be of great help in distinguishing pathological retinal changes from age-related ones.

In our study, we studied age-related changes in outer retinal layers' thickness and choroidal thickness in normal eyes aiming to identify the most influential factor on outer retinal layers' thickness.

Our results showed that macular CT was negatively correlated with age; this is in accordance with previous studies using EDI-OCT in normal eyes that have reported that macular choroidal thickness is negatively correlated with age [1, 5, 10, 23, 24].

Moreover, in our study, the thickness of RPE-OS showed a statistically significant negative correlation with age in the central foveal zone and also in the parafoveal and perifoveal rings, with a decline of 0.8 *μ*m per decade in the central 1 mm, when the entire cohort was compared. However, this number is just an impression of the theoretical speed of age-related changes based on the found linear relationship between thickness measurements and age and can only be verified with a longitudinal study. Regarding the ONL-IS, no statistically significant thinning was noticed with aging. This variable effect of aging on foveal layers may give a clue why earlier studies found no statistically significant correlations between age and overall central foveal retinal thickness, which is composed primarily of outer retinal layers [20, 21, 25].

Our findings are supported by earlier histological studies using fundus reflectometry that demonstrated loss of foveal cone visual pigment in the human retinae with aging [6, 26].

Our results are in concordance with a previous study by Abdolrahimzadeh et al. [22] who reported that, in all ETDRS grid zones, outer retinal layer thickness correlated positively with CT and negatively with age, also with Nieves-Moreno et al. [27] as they found negative correlation of the photoreceptor layer (measured as the thickness between the outer limiting membrane and Burch's membrane) with age in 3 ETDRS rings, and with Demirkaya et al. [8] who demonstrated a significant decrease in foveal outer segment layer (OSL) thickness with increasing age; however, they found that this decrease was nonsignificant in the perifoveal and parafoveal rings [8].

On the contrary, Bafik and his colleagues [5] reported that the outer retina did not show any age or sex-related differences; however, in their study, the outer retinal thickness was measured from the inner nuclear layer/outer plexiform layer (INL/OPL) junction to the outer border of the RPE layer; i.e., their measurement included RPE-OS and ONL-IS layers. This is unlike our study, where, we segregated the outer retina into layers, the outer one (RPE-OS) correlated with age while the inner one (ONL-IS) did not show any correlation with age. Won and coworkers [14] demonstrated that PHL, ONL, and foveal RPE thickness showed no significant differences with increasing age; however, their study only included 50 subjects which is a rather small sample, and the segmentation protocol they used is different from the one used in the present study since ONL, photoreceptors, and RPE are considered separately in the former which might be a possible reason for discrepancy.

By contrast, Ooto et al. [28] reported a thickening of the OS layer in all macular zones, unlike our results that showed a thinning in RPE-OS with aging. However, the RPE was not included in their segmentation algorithms. Worth mentioning that they stated in their discussion that RPE and OS tip lines were difficult to identify independently in some subjects; this ambiguity in the definition of the OS tips might have led to the different results. According to our device segmentation algorithm, we defined the RPE-OS layer as the layer between the IS/OS transition and outer surface of RPE, which are relatively clear transitions. Moreover, all subjects included in their study were Japanese adults, whereas foveal thickness differences between different ethnic groups have been previously reported [28].

Flores-Moreno et al. [29] did not find any correlation between age and photoreceptors-RPE layer thickness (measured from the hyper-reflective line corresponding to the ELM to the outer border of the RPE); this result may be due to the smaller number of patients (60) in their study, the selected group of highly myopic eyes (≥−6D) as opposed to our cohort where subjects with refractive error >+4 or <−4 were all excluded from the study, and also the different device used (Topcon 3D-2000 OCT).

Kenmochi et al. [30] stated that the RPE-COST thickness and the IS/OS-ELM thickness at the fovea were significantly associated with age. Conversely, they found that the COST-IS/OS thickness, which incorporates the outer segments of the photoreceptor cells, was not significantly associated with age. However, in their study, the RPE line and the COST line were sometimes not completely separated, as they described, and all their measurements relied on manual segmentation. In addition, all their measurements were taken from a single-line OCT scan and were reported as a single-point thickness, unlike our study where all measurements of outer retinal layers were done automatically in 12 radial lines and reported as thickness maps in the 9 ETDRS macular map sectors which reduce our measurements variability.

In this study, we found statistically significant positive correlation of RPE-OS with CT in all ETDRS macular grid zones, which agree with Abdolrahimzadeh et al. [22]. This was expected since the photoreceptor layer in the foveal region depends mainly in its nourishment on the underlying choroid. Furthermore, it has been previously reported that choroidal alterations and thinning in pathological conditions can lead to outer retinal layer thinning [29, 31].

In view of the fact that photoreceptor age-related loss might either be a consequence of age-related choriocapillary rarefaction (choroidal thinning) or age-related neural tissue loss, we performed a multiple regression analysis, in an attempt to understand the effects of age, choroid thickness, and other variables including gender, refraction, and axial length on the outer retinal layer thickness.

The multiple regression analysis revealed that the central foveal zone of RPE-OS as well as superior and inferior parafoveal and perifoveal zones was independently affected by age rather than choroidal thinning. This might suggest that the photoreceptor age-related neural tissue loss has more prominent impact than vascular supply diminution due to choroidal thinning on outer retinal layer thickness. Thus, it is essential to consider age when outer retinal layer thickness is evaluated in the context of monitoring the progression of outer retinal layer diseases like retinitis pigmentosa, evaluating the efficacy of various therapeutic modalities that target the photoreceptor layer and predicting visual prognosis in various macular diseases.

It is noteworthy that there is no general consensus on where to set the boundaries between retinal layers. This may partially explain the contrasting results obtained in different studies. In our study, we measured the RPE-OS layer (from the IS/OS junction to the outer aspect of RPE); however, thickening of the RPE with age has been previously reported [12, 27]. Accordingly, photoreceptors outer-segment thinning with age might be even greater than that reported in this study.

The strengths of the current study include our robust measurements of different layers. Measurements of outer retinal layers were based on automatic segmentation done by the device software (NAVIS-EX Image Filing software, RS3000-OCT, NIDEK, Gamagori, Japan) with no manual correction done which ensures the accuracy of the measurements. In addition, we measured thickness maps in 9 areas of 6 mm ETDRS macular grid rather than single-point foveal measurements on single-line OCT scans done in previous reports; where this central foveal zone is just a little portion of the retina and the information obtained from this area cannot be extended to all the other macular subfields. Even in other studies which measured parafoveal thickness, they relied on single-point thickness using single-line OCT scan which would be less accurate than the map we constructed. Regarding choroidal thickness, sclerochoroidal interface was manually drawn but with very good reliability measures, and the interface was drawn in each of the 12 radial lines of macular radial scan, and based on this, choroidal thickness map was generated; hence, our measurements are much more precise and show less variability. Finally, we had an appropriate group of normal individuals of wide-ranging age and properly distributed age groups.

Limitations of the present study include the cross-sectional design rather than the longitudinal design. Further studies with longitudinal data on a larger cohort are warranted and would aid in building up a normative database against which thickness maps of individual retinal layers in diseased eyes can be compared, which may advance our understanding of pathological mechanisms in various retinal degenerative diseases.

## Figures and Tables

**Table 1:** Demographic and ocular characteristics of the 3 studied groups' participants.

	Group 1 (mean ± SD)	Group 2 (mean ± SD)	Group 3 (mean ± SD)	*P*	G1 vs G2	G1 vs G3	G2 vs G3
Age, years	29.85 ± 4.9	49.22 ± 6.1	66.35 ± 4.1	**<0.001**	**<0.001**	**<0.001**	**<0.001**
Gender, M : F (*N*)	26 : 19	16 : 27	21 : 16	*X* ^2^ = 1.33 *P*=0.514	—	—	—
Refraction (spherical equivalent), diopters	−0.69 ± 0.61	−0.80 ± 0.59	−0.65 ± 0.62	0.640	0.530	0.744	0.358
Axial length (mm)	22.91 ± 0.37	23.02 ± 0.35	22.91 ± 0.28	0.251	0.141	0.994	0.164

Bold numbers refer to statistically significant values.

**Table 2:** Comparison of mean thickness (in *μ*m) of 9 ETDRS subfields for RPE-OS, ONL-IS, and choroid in the 3 studied groups.

	Group 1 (mean ± SD)	Group 2 (mean ± SD)	Group 3 (mean ± SD)	*P*	G1 vs G2	G1 vs G3	G2 vs G3
*RPE-OS*
Central RPE-OS	74.76 ± 3.0	74.63 ± 3.33	71.05 ± 4.81	**<0.001**	0.87	**<0.001**	**<0.001**
*Inner ring*							
Superior	67.62 ± 2.71	68.05 ± 3.28	65.19 ± 3.65	**<0.001**	0.54	**0.001**	**<0.001**
Inferior	67.73 ± 3.28	68.07 ± 3.48	64.84 ± 3.33	**<0.001**	0.64	**<0.001**	**<0.001**
Nasal	67.98 ± 2.77	67.56 ± 4.45	64.78 ± 4.11	**<0.001**	0.61	**<0.001**	**0.002**
Temporal	68.16 ± 2.99	68.98 ± 3.61	65.32 ± 3.57	**<0.001**	0.26	**<0.001**	**<0.001**
*Outer ring*							
Superior	65.04 ± 2.20	65.79 ± 3.44	62.46 ± 3.35	**<0.001**	0.25	**<0.001**	**<0.001**
Inferior	64.44 ± 2.34	64.256 ± 2.84	61.0 ± 3.46	**<0.001**	0.76	**<0.001**	**<0.001**
Nasal	64.91 ± 2.60	63.69 ± 4.46	61.24 ± 2.96	**<0.001**	0.10	**<0.001**	**0.002**
Temporal	64.60 ± 1.95	64.72 ± 3.62	61.54 ± 3.70	**<0.001**	0.86	**<0.001**	**<0.001**

*ONL-IS*
Central ONL-IS	83.98 ± 10.3	84.00 ± 24.57	88.16 ± 11.51	0.45	0.99	0.27	0.27
*Inner ring*							
Superior	69.69 ± 7.44	71.86 ± 8.68	69.29 ± 8.88	0.321	0.223	0.83	0.17
Inferior	69.16 ± 8.89	70.69 ± 9.218	71.51 ± 9.58	0.497	0.434	0.25	0.69
Nasal	70.62 ± 10.48	73.09 ± 10.88	68.57 ± 10.12	0.160	0.273	0.38	0.06
Temporal	69.38 ± 8.73	70.58 ± 8.79	75.00 ± 9.05	0.014	0.525	**0.005**	**0.03**
*Outer ring*							
Superior	61.13 ± 7.55	62.26 ± 8.46	61.621 ± 8.795	0.816	0.525	0.790	0.732
Inferior	57.18 ± 7.37	58.86 ± 8.55	59.540 ± 8.102	0.383	0.327	0.186	0.706
Nasal	58.53 ± 6.74	59.26 ± 9.53	58.757 ± 10.712	0.930	0.708	0.911	0.806
Temporal	60.51 ± 8.29	62.23 ± 8.77	64.324 ± 8.913	0.143	0.352	**0.049**	0.283

*CT*
Central CT	356.16 ± 59.88	296.21 ± 41.26	255.14 ± 33.61	**<0.001**	**<0.001**	**<0.001**	**<0.001**
*Inner ring*							
Superior	352.69 ± 56.17	291.442 ± 32.22	257.49 ± 33.04	**<0.001**	**<0.001**	**<0.001**	**0.001**
Inferior	345.73 ± 63.77	279.48 ± 32.06	254.97 ± 35.44	**<0.001**	**<0.001**	**<0.001**	**0.021**
Nasal	316.73 ± 50.67	263.14 ± 32.13	231.03 ± 33.68	**<0.001**	**<0.001**	**<0.001**	**0.001**
Temporal	338.80 ± 54.55	280.51 ± 35.21	243.70 ± 32.05	**<0.001**	**<0.001**	**<0.001**	**<0.001**
*Outer ring*							
Superior	338.07 ± 52.29	276.05 ± 28.84	244.86 ± 36.59	**<0.001**	**<0.001**	**<0.001**	**0.001**
Inferior	324.16 ± 61.13	258.49 ± 33.55	229.46 ± 51.99	**<0.001**	**<0.001**	**<0.001**	**0.011**
Nasal	273.18 ± 46.56	235.49 ± 36.32	207.73 ± 35.19	**<0.001**	**<0.001**	**<0.001**	**0.002**
Temporal	322.47 ± 52.19	261.88 ± 39.01	229.19 ± 31.94	**<0.001**	**<0.001**	**<0.001**	**0.001**

CT: choroidal thickness. Bold numbers refer to statistically significant values.

**Table 3:** Correlation of the 9 ETDRS subfields mean thickness of RPE-OS, ONL-IS, and CT with age, gender, refraction, axial length, and CT.

	Correlation with age		Correlation with gender		Correlation with CT		Correlation with refraction		Correlation with axial length	
	*r*	*P* value	*r*	*P* value	*r*	*P* value	*r*	*P* value	*r*	*P* value
*RPE-OS*
Central RPE-OS	−0.345	**<0.001**	−0.049	0.590	0.273	**0.002**	0.092	0.306	0.027	0.767
*Inner ring*										
Superior	−0.253	**0.004**	−0.119	0.186	0.182	**0.042**	0.069	0.446	−0.025	0.779
Inferior	−0.280	**0.002**	−0.100	0.268	0.227	**0.011**	−0.034	0.710	0.102	0.256
Nasal	−0.287	**0.001**	−0.062	0.492	0.339	**<0.001**	0.109	0.224	−0.047	0.601
Temporal	−0.260	**0.003**	−0.058	0.523	0.283	**0.001**	0.091	0.310	<0.001	0.997
*Outer ring*										
Superior	−0.264	**0.003**	−0.107	0.236	0.175	0.051	0.103	0.252	0.010	0.915
Inferior	−0.396	**<0.001**	−0.040	0.655	0.352	**0.001**	0.053	0.555	0.076	0.397
Nasal	−0.366	**<0.001**	−0.075	0.407	0.479	**<0.001**	0.164	0.068	−0.087	0.336
Temporal	−0.311	**<0.001**	−0.030	0.743	0.325	**<0.001**	0.071	0.429	0.010	0.915

*ONL-IS*
Central ONL-IS	0.08	0.376	0.019	0.833	−0.075	0.409	0.136	0.130	−0.124	0.167
*Inner ring*										
Superior	0.062	0.495	−0.173	0.054	−0.024	0.791	−0.012	0.893	−0.001	0.994
Inferior	0.143	0.111	−0.185	0.039	−0.047	0.599	0.005	0.953	0.017	0.853
Nasal	−0.046	0.614	−0.132	0.142	0.114	0.207	0.094	0.297	0.123	0.171
Temporal	0.294	**0.001**	−0.095	0.292	−0.237	**0.008**	−0.037	0.681	−0.047	0.604
*Outer ring*										
Superior	0.073	0.417	−0.099	0.271	−0.039	0.665	0.027	0.766	−0.016	0.861
Inferior	0.164	0.068	−0.144	0.109	−0.124	0.168	−0.032	0.725	0.066	0.463
Nasal	0.057	0.530	−0.082	0.361	−0.114	0.206	0.004	0.962	−0.030	0.739
Temporal	0.222	**0.013**	−0.014	0.120	−0.135	0.133	−0.071	0.433	−0.014	0.880

*CT*
Central CT	−0.725	**<0.001**	−0.013	0.255	—	—	0.166	0.064	−0.015	0.868
*Inner ring*										
Superior	−0.727	**<0.001**	−0.101	0.260	0.865	**<0.001**	−0.003	0.976	0.054	0.547
Inferior	−0.669	**<0.001**	−0.120	0.183	0.837	**<0.001**	0.015	0.871	0.057	0.528
Nasal	−0.672	**<0.001**	−0.119	0.186	0.784	**<0.001**	−0.030	0.744	0.017	0.853
Temporal	−0.683	**<0.001**	−0.110	0.224	0.781	**<0.001**	−0.018	0.840	−0.024	0.790
*Outer ring*										
Superior	−0.729	**<0.001**	−0.072	0.424	0.817	**<0.001**	−0.003	0.976	0.065	0.470
Inferior	−0.651	**<0.001**	−0.142	0.114	0.772	**<0.001**	0.045	0.621	0.075	0.403
Nasal	−0.566	**<0.001**	−0.177	0.048	0.700	**<0.001**	0.000	0.998	0.054	0.549
Temporal	−0.658	**<0.001**	−0.094	0.296	0.704	**<0.001**	0.020	0.822	−0.047	0.601

CT: choroidal thickness. Bold numbers refer to statistically significant values.

**Table 4:** Multiple regression analysis of different factors affecting the thickness of 9 ETDRS subfields of RPE-OS and ONL-IS.

	Regression with age		Regression with gender		Regression with CT		Regression with refraction		Regression with axial length	
	*B*-coefficient	*P* value	*B*-coefficient	*P* value	*B*-coefficient	*P* value	*B*-coefficient	*P* value	*B*-coefficient	*P* value
*RPE-OS*
Central RPE-OS	−0.087	**0.010**	−0.664	0.355	0.001	0.879	0.691	0.218	0.213	0.838
*Inner ring*										
Superior	−0.065	**0.023**	−1.059	0.088	−0.003	0.716	0.472	0.327	−0.304	0.729
Inferior	−0.060	**0.031**	−0.793	0.227	0.002	0.777	−0.067	0.895	0.926	0.319
Nasal	−0.032	0.297	−0.452	0.525	0.019	**0.033**	0.778	0.161	−0.561	0.578
Temporal	−0.033	0.256	−0.438	0.510	0.012	0.135	0.610	0.240	0.070	0.941
*Outer ring*										
Superior	−0.070	**0.011**	−0.989	0.095	−0.005	0.547	0.658	0.158	0.088	0.918
Inferior	−0.064	**0.007**	−0.307	0.586	0.007	0.230	0.310	0.479	0.557	0.483
Nasal	−0.036	0.120	−0.284	0.641	0.031	**<0.001**	0.960	**0.042**	−1.107	0.196
Temporal	−0.039	0.128	−0.212	0.727	0.012	0.081	0.404	0.396	0.182	0.834

*ONL-IS*
Central ONL-IS	0.083	0.568	0.204	0.948	0.001	0.985	3.383	0.169	−5.519	0.227
*Inner ring*										
Superior	0.022	0.753	−2.866	0.067	−0.001	0.939	0.112	0.926	−0.059	0.979
Inferior	0.090	0.211	−3.189	0.063	0.005	0.786	0.390	0.769	0.481	0.786
Nasal	−0.181	**0.027**	−4.218	**0.029**	−0.063	**0.010**	2.045	0.171	3.819	0.160
Temporal	0.127	0.075	−1.601	0.329	−0.016	0.418	−0.483	0.705	−1.234	0.597
*Outer ring*										
Superior	0.037	0.604	−1.622	0.291	0.001	0.965	0.492	0.683	−0.320	0.884
Inferior	0.052	0.401	−2.344	0.119	−0.011	0.493	−0.063	0.957	1.685	0.424
Nasal	−0.023	0.721	−2.065	0.226	−0.029	0.174	0.241	0.854	−0.649	0.786
Temporal	0.116	0.085	−2.082	0.192	−0.001	0.943	−0.811	0.515	−0.368	0.943

CT: choroidal thickness. Bold numbers refer to statistically significant values.

## Data Availability

The data used to support the findings of this study are available from the corresponding author upon request.
